# Modified Dual Docking Robotic Surgery for Hereditary Paraganglioma-Pheochromocytoma Syndrome

**DOI:** 10.7759/cureus.16947

**Published:** 2021-08-06

**Authors:** Chen-Yueh Wen, Chia-Mu Tsai, Chia-Cheng Yu, Jen-Tai Lin

**Affiliations:** 1 Division of Urology, Department of Surgery, Kaohsiung Veterans General Hospital, Kaohsiung, TWN

**Keywords:** robotic surgery, hereditary paraganglioma-pheochromocytoma syndrome, adrenalectomy, partial cystectomy, palpitation

## Abstract

Hereditary paraganglioma-pheochromocytoma (PGL/PCC) syndrome is an uncommon genetic condition featured by an inherited predisposition to generate PGLs. Surgical resection of all tumors is the standard treatment for excess adrenaline production and tendency for metastasis. Nowadays, there are few case reports that have mentioned the surgical technique for hereditary PGL/PCC syndromes, especially robot-assisted surgery. Herein we present a rare case of hereditary PGL/PCC syndromes treated by partial cystectomy and right adrenalectomy at the same time with modified dual docking robotic surgical technique. Our dual docking robotic technique is a feasible option for patients with hereditary PGL/PCC syndromes of synchronous tumors in bladder and adrenal gland. It could not only prevent from second surgery but be safely performed without compromising disease control.

## Introduction

The hereditary paraganglioma-pheochromocytoma (PGL/PCC) syndrome is a collection of autosomal-dominant hereditary cancer diseases, which tend to generate PGLs [1]. Surgical resection of all tumors in cases of hereditary PGL/PCC syndromes is the standard treatment for excess adrenaline production and is important because of tendency for metastasis [2,3]. However, due to the relatively uncommon PGL/PCC syndromes of synchronous tumors, there are few case reports that have mentioned the surgical technique, especially robot-assisted surgery. Herein we present a rare case of hereditary PGL/PCC syndromes of synchronous tumors in bladder and right adrenal gland treated by partial cystectomy and right adrenalectomy at the same time with dual docking robotic surgical technique.

## Case presentation

**Figure 1:**
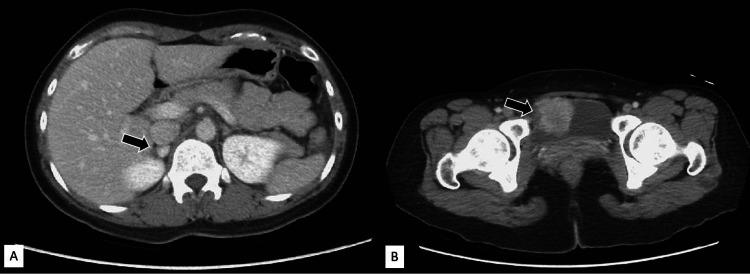
Axial computerized tomography sections reveal (A) a 1-cm mass (arrow) in the right adrenal gland and (B) a 1.7 cm × 2.4 cm mass (arrow) over the anterior wall of the bladder

A 32-year-old woman was incidentally found to have a urinary bladder mass due to epigastric pain for five months. Abdominal computed tomography demonstrated a mass up to 4.5 cm over anterior wall of the bladder and a nodule arising from right adrenal gland (Figure 1). Transurethral resection of the bladder tumor was performed uneventfully and pathology confirmed the diagnosis of bladder PGL with bladder muscular invasion. Therefore, she was prompted a referral to our institution for further management. Tracing back history, episodes of sudden onset of palpitations, chest tightness, and headache after voiding were noted on and off over the past six years.

**Figure 2:**
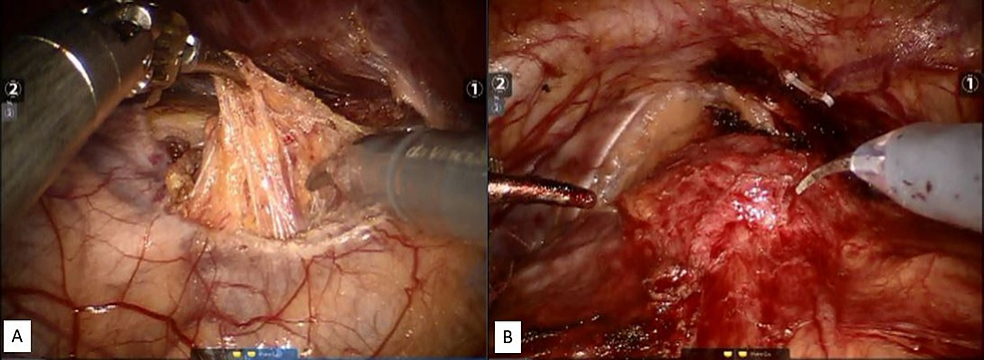
Operative images taken during the robotic surgery. (A) Right adrenal tumor with exposed upper pole of right kidney and suprarenal vein. (B) Bladder tumor

We advised her to receive robotic partial cystectomy and right adrenalectomy simultaneously. We used the Da Vinci surgical system Si® (Intuitive Surgical Sunnyvale, CA, USA) with a transperitoneal approach. First, the patient was placed in a modified lithotomy with steep Trendelenburg position for conducting partial cystectomy. The space of Retzius was developed and bladder was dissected from the anterior abdominal wall by dividing the urachus. We used monopolar electrocautery to incise the peritoneum overlying the bladder mass. It was carefully dissected from the retroperitoneal structures and circumferentially resected from the bladder. The defect was closed with 2-0 V-Loc sutures. Then, she was placed in modified lateral decubitus position. Gerota's fascia was incised at the junction of the upper pole of right kidney and adrenal gland. The perirenal fat was dissected freely from psoas muscle and peritoneum to approach the adrenal tumor. Finally, it was removed en-bloc along with the right adrenal gland (Figure 2). The docking times for both procedures were 145 minutes and 80 minutes, respectively. Total blood loss was 50 ml. No immediate intraoperative complication was noted. Convalescence after robotic surgery was fair and the duration of hospital stay was four days.

**Figure 3:**
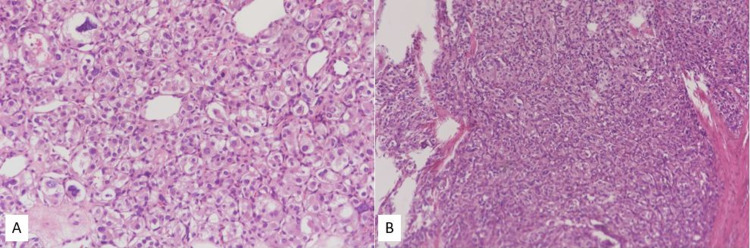
The tumor cells reveal prominent Zellballen pattern, composed of round to oval cells with abundant granular eosinophilic cytoplasm, hematoxylin and eosin, (A) adrenal gland ×20, (B) urinary bladder x10

**Figure 4:**
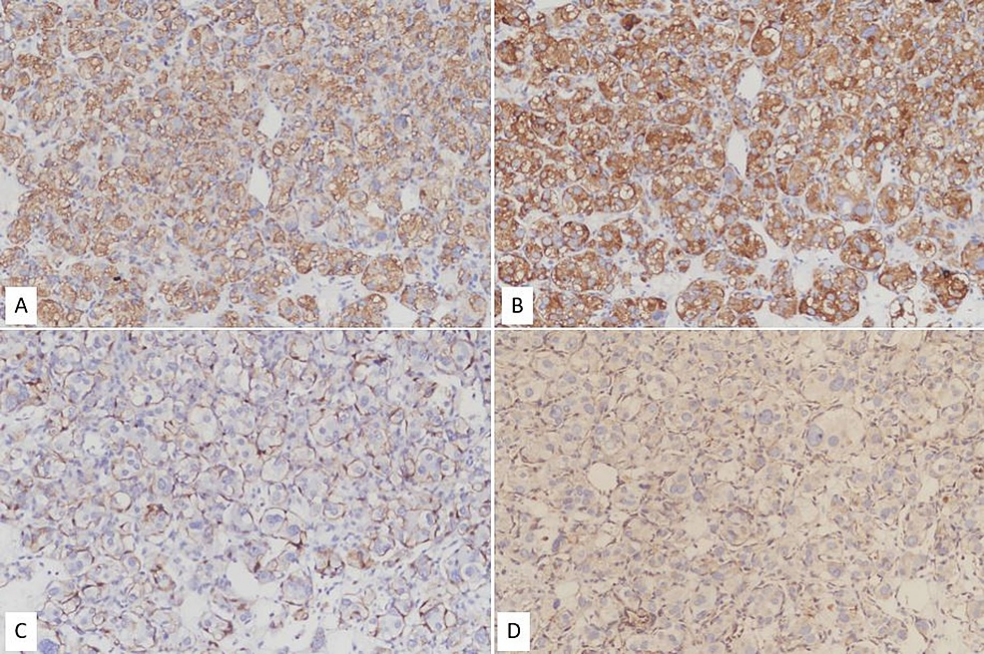
The tumor cells are positive for (A) chromogranin-A, (B) synaptophysin, and (C) S100 and negative for (D) SDHB (immunohistochemical stain, ×20) SDHB, succinate dehydrogenase B

The final pathological report revealed PCC of right adrenal gland and PGL of urinary bladder. The tumor cells revealed prominent Zellballen pattern, composed of round to oval cells with abundant granular eosinophilic cytoplasm (Figure 3). S100 positive sustencular cells surrounding each nest of tumor cells were present. Immunohistochemical study revealed the tumor cells positive for chromogranin-A and synaptophysin and negative for CK AE1/AE3. Further immunohistochemical stain with succinate dehydrogenase B (SDHB) was performed for surveillance due to synchronous existence of tumors, which revealed negative result of the tumor cells (Figure 4). The above result may suggest the presence of a germline pathogenic variant of the SDH subunits, which is compatible with hereditary PGL/PCC syndrome caused by SDHx mutations. Epigastric discomfort and sympathetic symptoms improved significantly after operation. No recurrence was seen on follow-up abdominal computed tomography. 

## Discussion

Hereditary PGL/PCC syndromes are neuroendocrine tumors that arise from chromaffin cells in the adrenal gland (PCC) or in extra-adrenal sympathetic nerve ganglia (PGL) [4]. The incidence of hereditary PGL/PCC syndromes is not precisely known as reported by Else et al. [5]. On the other hand, the incidence of PCC is approximately 0.6/100,000 per year and PGL is lower [6]. About 25% of all PCCs arise in individuals with a hereditary predisposition and PGLs have higher association with hereditary predisposition. Overall, 35%-40% of all PGL/PCC syndromes are associated with a hereditary predisposition [5].

Treatment of hereditary PGL/PCC syndrome includes a variety of management, such as active surveillance, catecholamine blockade, surgery, chemotherapy, and radiation therapy, while surgical removal remains the mainstream standard treatment for localized or synchronous tumors [2,7]. Transurethral resection of bladder PGL is an alternative management, but complete removal of the tumor is considered difficult and it may precipitate a hypertensive crisis or arrhythmia perioperatively. Many surgeons advocate operations with open, laparoscopic, or robotic methods for bladder PGL or adrenal PCC. Compared to open surgery, laparoscopic partial cystectomy or adrenalectomy offers the advantage of a smaller incision, minimally invasive procedure, less short-term postoperative pain, and a faster convalescence [8]. The benefits of robotic surgery have increased with being capable of the three-dimensional vision, articulating instruments, superior dexterity, and stable surgical platform. In our case, we performed robotic partial cystectomy and right adrenalectomy simultaneously via dual docking of the robot, which could prolong surgical time. Robots may allow surgeons to feel more comfortable owing to ergonomic design. The patient did not encounter intraoperative hypertension or arrhythmia when manipulating tumors. It reminds us of the use of dual docking robotic technique as secure for patient with synchronous PGL/PCC and preventing from two-stage surgery.

We acknowledge that our case is lack of long-term follow-up and further studies also consider that these patients with hereditary PGL/PCC syndromes of synchronous tumors in bladder and adrenal gland are necessary for providing further genetic information. Hence, we should refer our patients for genetic test due to pathogenic variants in suspicious genes.

## Conclusions

To the best of our knowledge, this is the first case report of successfully treating hereditary PGL/PCC syndrome via dual docking robotic partial cystectomy and right adrenalectomy. Dual docking robotic technique is a feasible option for patients with hereditary PGL/PCC syndromes of synchronous tumors in bladder and adrenal gland. It could not only prevent from second surgery but be safely performed without compromising disease control.
